# Advanced ECG feature extraction and SVM classification for predicting defibrillation success in OHCA

**DOI:** 10.3389/fcvm.2025.1550422

**Published:** 2025-07-16

**Authors:** Haqi Zhang, Xiaotian Pan, Shan Zhou, Weiwei Zhang, Jing Chen, Limin Pan

**Affiliations:** ^1^ Institute of Intelligent Media Computing, Hangzhou Dianzi University, Hangzhou, China; ^2^ Shangyu Institute of Science and Engineering Co. Ltd., Hangzhou Dianzi University, Shaoxing, China; ^3^ Department of Ultrasound, The Affiliated Huaian No. 1 People’s Hospital of Nanjing Medical University, Huaian, China; ^4^ Infectious Disease Department, The Third Affiliated Hospital of Wenzhou Medical University, Wenzhou, China; ^5^ Department of Anesthesiology, The Third Affiliated Hospital of Wenzhou Medical University, Wenzhou, China

**Keywords:** out-of-hospital cardiac arrest (OHCA), electrocardiogram (ECG), defibrillation outcome prediction, machine learning, support vector machine (SVM), feature selection

## Abstract

Out-of-hospital cardiac arrest (OHCA) represents a critical challenge for emergency medical services, with the necessity for rapid and accurate prediction of defibrillation outcomes to enhance patient survival. This study leverages a dataset of 251 ECG signals from OHCA patients, consisting of 195 unsuccessful and 56 successful resuscitation attempts as categorized by expert cardiologists. We extracted six crucial features from each ECG signal: heart rate, QRS complex amplitude, QRS complex duration, total power, low-frequency power (0.04–0.15 Hz), and high-frequency power (0.15–0.4 Hz). These features were derived using standard temporal and frequency domain methods. Subsequent analysis focused on selecting the most predictive features, with QRS complex amplitude, total power, and low-frequency power showing the highest discriminative ability based on their Area Under the Curve (AUC) values. A Support Vector Machine (SVM) classifier, trained on these selected features, demonstrated a prediction accuracy of 95.6%, highlighting the efficacy of combining targeted ECG signal features with machine learning techniques to forecast defibrillation success accurately. This approach provides a non-invasive, rapid, and reliable method to support clinical decisions during OHCA emergencies. Future research aims to expand the dataset, refine feature extraction techniques, and explore additional machine learning models to further enhance prediction accuracy. This study underscores the potential of ECG-based feature analysis and targeted machine learning in improving resuscitation strategies, ultimately contributing to higher survival rates in OHCA patients.

## 1 Introduction

Out-of-hospital cardiac arrest (OHCA) is a critical medical emergency with high mortality rates, necessitating immediate and effective intervention to increase the chances of survival. The ability to predict the outcome of defibrillation attempts in real-time can significantly enhance resuscitation strategies and improve patient outcomes. Electrocardiogram (ECG) signals, which provide valuable insights into the electrical activity of the heart, are essential for monitoring and diagnosing cardiac conditions during such emergencies. By leveraging advanced signal processing techniques and machine learning algorithms, it is possible to analyze ECG data and extract meaningful features that can predict the success of resuscitation efforts. The predominant initial rhythm in cases of OHCA is identified as ventricular fibrillation (VF), which is marked by rapid, chaotic heart muscle contractions and ECG waveforms that are typically unrecognizable. The administration of a controlled electrical current to the fibrillating heart through electrical defibrillation remains the sole effective intervention for reversing VF and reinstating orderly electrical activity (1). Despite evidence supporting the advantages of early defibrillation in enhancing survival rates following observed cardiac arrests (2, 3), the benefits of immediate defibrillation in prolonged VF cases remain contentious. The probability of a successful defibrillation declines swiftly as the duration of untreated VF extends, largely due to increased oxygen demands by the myocardium during prolonged VF, leading to energy depletion and acidosis, which complicate the restoration of normal heart activity (4). Conversely, initiating cardiopulmonary resuscitation (CPR) can improve myocardial conditions. Clinical data shows a significant reduction in survival rates—7%–10% per minute—for VF cases not immediately treated, while prompt and effective CPR reduces this rate to only 3%–4% per minute (5). Research has highlighted a critical juncture at approximately 4–5 min where starting CPR prior to defibrillation significantly enhances the likelihood of reverting to normal cardiac function (6, 7). However, pinpointing the exact onset of VF during OHCA often proves impossible, complicating the determination of treatment priorities between CPR and immediate defibrillation. Additionally, repeated failed attempts at defibrillation with high energy can exacerbate damage to the already compromised myocardium and can lead to the deterioration of VF into conditions like asystole, which are notoriously difficult to treat (8). Consequently, the precise timing of defibrillation, guided by predictions of shock outcomes, has garnered considerable interest. Modern automated external defibrillators, which routinely analyze ECG waveforms, provide a critical tool for guiding resuscitation efforts based on patient conditions. Assessing the likelihood of successful defibrillation outcomes allows for the optimal timing of shocks to be determined, advocating for the avoidance of shocks when the probability of success is low and instead recommending the use of CPR and chest compressions (9). This strategy minimizes unnecessary defibrillation attempts, potentially offering greater benefits than a uniform treatment approach for all VF patients. Over the past two decades, various strategies utilizing singular or combined VF features derived from pre-shock ECG episodes have been employed to predict outcomes of defibrillation attempts (10). Notably, only one study (10) has addressed the issue of class imbalance by employing cost-sensitive classification to assess the likelihood of successful defibrillation, an essential consideration since many machine learning algorithms aim to optimize overall accuracy but may perform poorly on imbalanced datasets (11, 12). A key study found the optimal feature subset for detecting shockable rhythms with sensitivity and specificity of over 94% (13). The researchers utilized short ECG segments to calculate 30 features and trained multiple machine learning models, including logistic regression, bagging, random forest, boosting, and support vector machines. Similarly, convolutional neural networks have been applied to automatically extract significant features and perform classifications, achieving impressive accuracies and demonstrating the potential of deep learning algorithms in this field (14). Although these findings are promising, merely distinguishing between shockable and non-shockable rhythms does not suffice for optimizing defibrillation timing. A deeper understanding of the factors predicting successful shock outcomes is crucial. Several studies have shown mixed results when using various feature combinations to predict defibrillation success; some did not find any improvement over using single features (15, 16). In prior studies such as reference (15), investigations into the effectiveness of combining multiple ECG features to predict defibrillation outcomes have shown mixed results. Those studies concluded that integrating a variety of predictive features derived from ECG data does not necessarily enhance predictive accuracy, often failing to significantly exceed an 87% threshold. This has led to a reevaluation of the predictive strategies employed in cardiac arrest management, suggesting that a more selective approach might yield better results. This backdrop sets the stage for our study, where we aim to refine the selection of ECG features and focus on those with the most substantial impact on prediction outcomes. Moreover, using principal component analysis to enhance outcome predictions by combining wavelet-based features has not proven effective, as the technique focuses on maximizing variance rather than improving class distinction (17). However, improvements were noted when two decorrelated PCA features were combined (18). Further studies employing linear discriminant analysis on multiple features demonstrated enhanced predictions (19), and genetic programming using a small set of features showed potential for reducing unsuccessful defibrillations (20). Systematically optimized support vector machine (SVM) algorithms employing embedded 10-fold cross-validation with a combination of features outperformed single-feature models in terms of precision and area under the curve, demonstrating the value of advanced modeling techniques in healthcare settings (10). Reference (21) provided a foundational approach to predicting defibrillation outcomes using SVM models, utilizing a dataset of only 41 patients. The restricted size of the patient cohort in this study raises significant concerns regarding the model's ability to generalize across a broader population. Additionally, they employed standard waveform features such as AMSA, slope, and RMS to predict defibrillation outcomes, achieving a modest accuracy of approximately 81%. Due to the retrospective nature of the ECG dataset, information on sociodemographic variables such as age, sex, or ethnicity was not available, limiting our ability to assess potential health inequalities across groups. Future studies should incorporate diverse patient data to evaluate model fairness and address disparities in OHCA outcomes.

The proposed SVM-based prediction model is designed to assist emergency physicians and paramedics in out-of-hospital cardiac arrest (OHCA) scenarios by predicting defibrillation success, enabling informed decisions on whether to prioritize defibrillation or cardiopulmonary resuscitation (CPR) within the acute care pathway.

This study employs feature extraction and machine learning techniques, specifically utilizing a Support Vector Machine (SVM) classifier, to forecast whether a patient will be successfully resuscitated. Here are the major contributions of current paper:

Extraction of New Features from ECG Signals: We have developed and extracted new, innovative features from ECG signals, which are designed to provide a deeper and more nuanced understanding of the physiological states associated with defibrillation outcomes.

Selection of Effective and Useful Features for SVM Model Application: Our research has identified and selected the most effective and informative features for application in the SVM model, ensuring that the model is both robust and sensitive to the critical nuances in the data.

Improved Accuracy in Prediction: The integration of these new features and the optimized selection process have significantly enhanced the accuracy of our SVM model in predicting defibrillation outcomes, demonstrating substantial improvements over existing methods.

The structure of this paper is as follows: after the introduction, we detail the data collection and preprocessing methods, followed by the feature extraction process. Next, we describe the machine learning approach used to develop the predictive model. Finally, we present the results and discussions, and conclude with the implications of our findings and potential directions for future research.

## 2 Data acquisition

The ECG signals were sourced from a dataset by Benini et al. (22), comprising 260 recordings from out-of-hospital cardiac arrest (OHCA) patients treated by emergency medical services. Out of the 260 ECG signals, 56 were identified as successful resuscitation cases (Return of Organized Electrical Activity, ROEA), 195 as unsuccessful resuscitation cases (No Return of Organized Electrical Activity, NoROEA), and 9 cases were indeterminable and excluded from further analysis (22). Each signal includes a 9-second pre-shock waveform and a 1-minute post-shock waveform, recorded using a semiautomatic Heartstart 3000 defibrillator (Laerdal Medical, Stavanger, Norway) with a standard lead II configuration. For preprocessing, as described in (22), the 9-second pre-shock episodes were uniformly resampled to 250 Hz and filtered using a 0.5–48 Hz bandpass filter to suppress residual baseline drift (frequencies below 0.5 Hz), power line interference (50/60 Hz), and high-frequency noise (e.g., muscle artifacts above 48 Hz). The 0.5–48 Hz frequency range was chosen to preserve the key morphological and spectral components of ventricular fibrillation (VF) waveforms, which typically lie between 1 and 10 Hz, ensuring suitability for feature extraction and machine learning analysis (23). Although (22) does not specify the filter type or order, a 4th-order Butterworth bandpass filter was implemented, as it is widely used in ECG preprocessing for its maximally flat passband response, which minimizes signal distortion while effectively rejecting noise (23). The filter's transfer function is given in Equation 1:

(1) $$H(z)=\frac{b_{0}+b_{1}z^{-1}+b_{2}z^{-2}+\ldots +b_{M}z^{-M}}{1+a_{1}z^{-1}+a_{2}z^{-2}+\ldots +a_{N}z^{-N}}$$

where *b_i_* and *a_i_* are the filter coefficients determined based on the cutoff frequencies (0.5 and 48 Hz) and the sampling rate (250 Hz). The filter was applied digitally, consistent with the digitization process using FindGraph software described in (22). To ensure reproducibility, a MATLAB implementation of the resampling and filtering steps is provided in Appendix.

**Figure 1:**
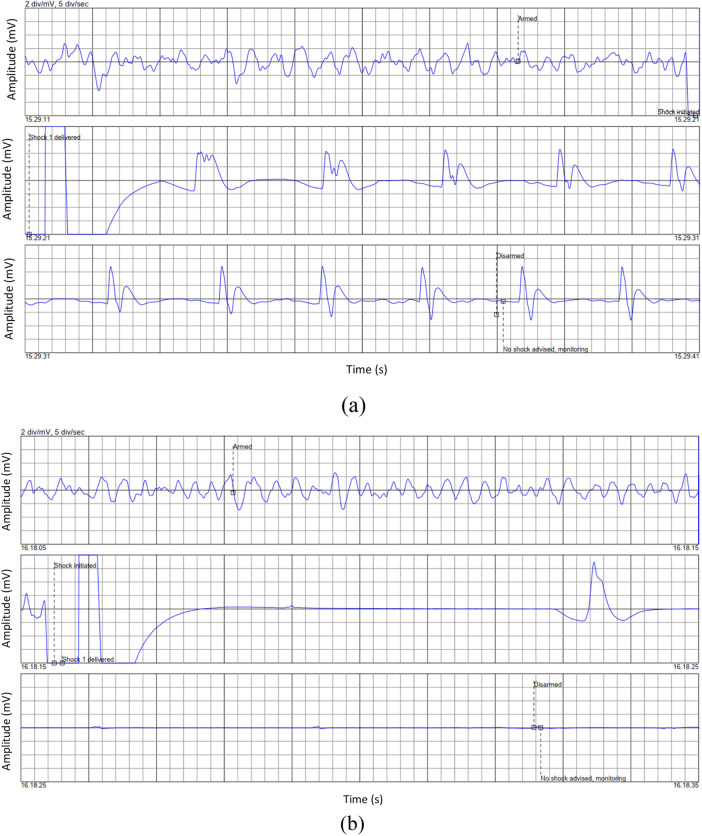
Representative ECG waveforms prior to defibrillation in: **(a)** successful, and **(b)** unsuccessful resuscitation outcomes (22).

Figure 1 in their paper illustrates the ECG waveform for two cases: one where resuscitation was successful (ROEA) and one where resuscitation was not successful (NoROEA), This figure displays ECG traces from two different cases captured immediately before defibrillation attempts. Panel (a) shows the ECG waveform of a patient where resuscitation was successful (ROEA), and panel (b) represents a patient where resuscitation was not successful (NoROEA). Each panel is divided into three sections:

First Section (Armed): Indicates the state of the defibrillator being armed in preparation for a potential shock, capturing the heart's rhythm and condition just before intervention.

Second Section (Shock Administered/Shock Not Administered): Depicts the moment a shock was administered in successful cases or withheld due to assessment findings in unsuccessful cases, showing the immediate response or non-response of the heart.

Third Section (Post-Shock): Shows the post-defibrillation ECG, which either returns to a stable rhythm in successful resuscitations or remains in a critical state in unsuccessful attempts.

These ECG segments highlight the critical differences in cardiac electrical activity and response under varying conditions, providing insights into the factors that may influence the outcome of resuscitation efforts.

Patients were included if they experienced OHCA with an initial rhythm of ventricular fibrillation (VF) and had a 9-second ECG signal recorded immediately prior to defibrillation. Exclusion criteria included incomplete ECG recordings or cases with non-VF rhythms.

The study population consisted of 251 patients with out-of-hospital cardiac arrest (OHCA) who had 9-second ECG signals recorded prior to defibrillation attempts, with 56 (22.3%) achieving successful resuscitation and 195 (77.7%) experiencing unsuccessful resuscitation, as determined by expert cardiologists. Due to the retrospective nature of the dataset, demographic characteristics such as age, sex, or comorbidities were not available, limiting the ability to describe the population's diversity.

## 3 Data processing

The objective of feature extraction from ECG signals is crucial in predictive modeling, particularly for the prediction of outcomes from cardiac arrest scenarios (24–26). This study emphasizes extracting meaningful features from 9-second ECG recordings immediately preceding defibrillation efforts. The extracted features aim to distinguish between successful (ROEA) and unsuccessful (NoROEA) resuscitations with high accuracy. The features extracted and analyzed in this study include:

Heart Rate (HR): Calculated from the average interval between successive QRS complexes noted in the ECG trace. It provides insights into the heart's rhythm just before defibrillation. The formula used is shown in Equation 2:

(2) $$\mathrm{HR}=\frac{60}{\mathrm{mean}\;\;\mathrm{RR}\;\mathrm{interval}}$$

where the RR interval denotes the duration between consecutive heartbeats.

QRS Complex Amplitude (amplitude_QRS): This feature measures the amplitude difference between the peak and trough of the QRS complex, which is shown in Equation 3, reflecting the electrical activity during ventricular contraction:

(3) amplitude_QRS=max(QRS)−min(QRS)

Total Power (total_power2): Represents the overall energy within the ECG signal frequency spectrum. This parameter, which is shown in Equation 4, is crucial for understanding the general electrical activity of the heart prior to a shock:

(4) $$\mathrm{total}\_\mathrm{power}2=\int X{\left(f\right)}^{2}\mathrm{df}$$

where *X*(*f*) is the Fourier transform of the ECG signal.

Low Frequency Power (lf_power2): As shown in Equation 5, it indicates the power in the low-frequency band (0.04–0.15 Hz), typically associated with autonomic nervous system modulations:

(5) $$\mathrm{If}\_\mathrm{power}2=\int_{0.04}^{0.15}X{\left(f\right)}^{2}\mathrm{df}$$

High Frequency Power (hf_power2): As shown in Equation 6, it represents the power in the high-frequency band (0.15–0.4 Hz), relevant to capturing fast, minute variations in heart rate variability:

(6) $$\mathrm{hf}\_\mathrm{power}2=\int_{0.15}^{0.4}X{\left(f\right)}^{2}\mathrm{df}$$

**Figure 2:**
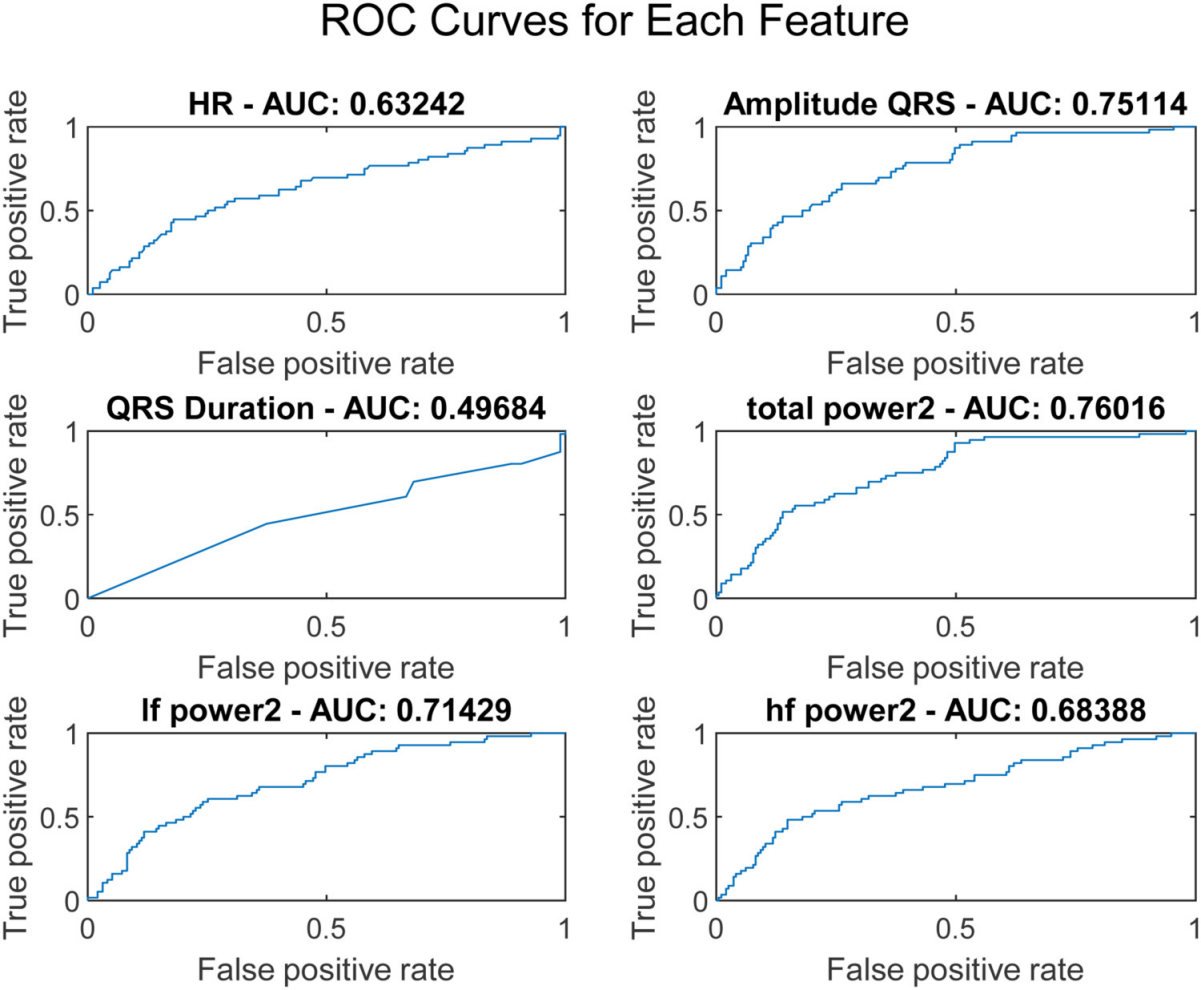
ROC curves for each feature extracted from 9-second pre-defibrillation ECG signals.

QRS Duration: The duration of the QRS complex, as shown in Equation 7, measured in milliseconds, provides insights into the time taken for ventricular depolarization:

(7) QRSDuration=t(max(QRS))−t(min(QRS))

where *t* represents the time corresponding to the maximum and minimum points of the QRS complex. To assess the discriminative capability of each feature, Receiver Operating Characteristic (ROC) curves were plotted, as illustrated in Figure 2.

No missing data were identified in the ECG dataset, as all 251 signals were complete 9-second recordings suitable for feature extraction.

**Figure 3:**
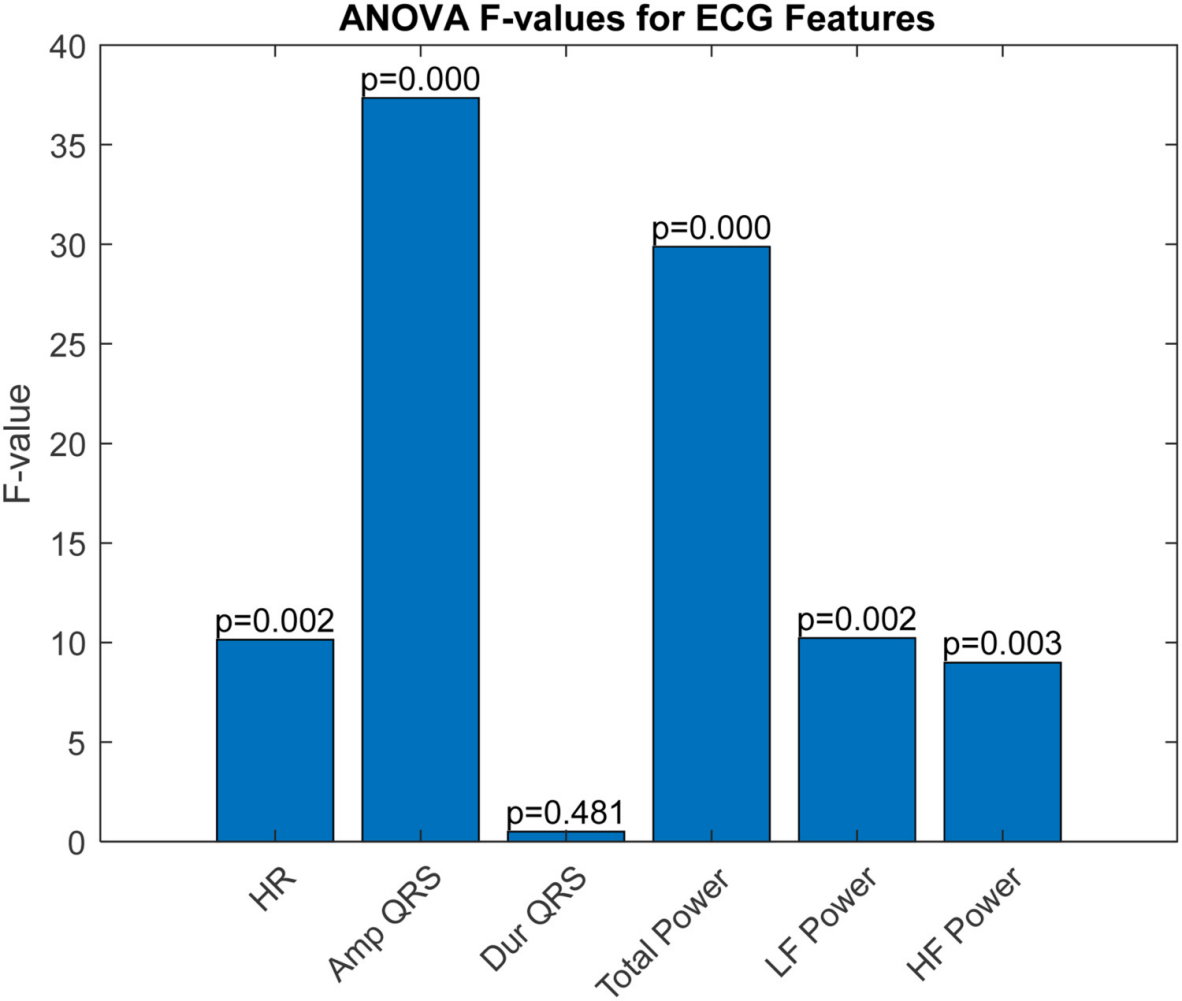
Analysis of variance (ANOVA) *F*-values and *P*-values for ECG features.

The ROC curve is a graphical representation that shows the performance of a diagnostic test across all classification thresholds, plotting the true positive rate (sensitivity) against the false positive rate (1-specificity). The Area Under the Curve (AUC) is derived from the ROC curve and quantifies a classifier's ability to differentiate between classes. The AUC value ranges from 0 to 1, where 1 indicates perfect classification and 0.5 indicates a performance no better than chance. Analysis of Variance (ANOVA) is a statistical method used to determine the presence of significant differences between the means of three or more independent (unrelated) groups. By decomposing variability within and between groups, ANOVA provides a robust test for assessing the relative significance of different categorical factors on a continuous outcome variable. In our study, ANOVA was employed to further validate the discrimination efficacy of six ECG features identified as potentially predictive in initial analyses. The results, illustrated in Figure 3, perform a critical role in substantiating the selection of amplitude_QRS, total_power2, and if_power. Figure 3 presents the ANOVA *F*-values for each of the six studied ECG features. This figure effectively highlights the statistical strength and discriminative power of the features within our analysis. amplitude_QRS and total_power2 showed the highest *F*-values, indicating a robust ability to distinguish between the groups under study. These features, along with lf_power2, which also demonstrated a significant *F*-value, were corroborated by the ANOVA as having a substantial impact in differentiating between successful and unsuccessful resuscitations. The clear disparities in *F*-values and low *P*-values confirm the reliability of these measurements in our predictive models, reinforcing the importance of these features in clinical assessments and decision-making processes related to cardiac arrest interventions.

These features not only exhibited superior AUC values but also significant ANOVA *F*-values, confirming their statistical significance and practical utility in differentiating outcomes in our dataset. Based on the AUC and ANOVA analysis, amplitude_QRS, total_power2, and lf_power2 were chosen as inputs for the SVM model. These features capture significant electrical and physiological changes in the heart that are critical for predicting the outcome just before defibrillation. Their combination offers a potent mix for effective and accurate predictive modeling, enhancing the model's ability to classify the resuscitation outcomes efficiently.

## 4 Support vector machine (SVM)

Support Vector Machines (SVMs) are a class of supervised learning algorithms used for classification tasks. SVMs are particularly effective in high-dimensional spaces and are known for their robustness and efficiency in handling both linear and non-linear data. The core idea behind SVM is to find a hyperplane that best separates the data points of different classes. For a binary classification problem, SVM aims to find the optimal hyperplane that maximizes the margin between two classes. The margin is defined as the distance between the closest points (support vectors) of each class to the hyperplane. The mathematical formulation of the SVM can be described as follows (27, 28):

Given a set of training examples $\{x_{i}.y_{i}{\}}_{i=1}^{n}$, where $x_{i}\in \;R^{d}$ and $y_{i}\in \{-1.1\}$, the objective is to solve the following optimization problem shown in Equation 8:

(8) $$\min_{w.b}\frac{1}{2}\|w{\|}^{2}\;\mathrm{subject}\;\;\mathrm{to}\;y_{i}(w\cdot x_{i}+b)\geq 1\;\;i=1.\;\ldots .n$$

here, *w* is the weight vector, and *b* is the bias term. This optimization problem can be solved using Lagrange multipliers, leading to the dual form of the problem shown in Equation 9:

(9) $$\max_{\alpha }\sum_{i=1}^{n}\alpha_{i}-\frac{1}{2}\sum_{i=1}^{n}\sum_{\;j=1}^{n}\alpha_{i}\alpha_{j}y_{i}y_{j}(x_{i}\cdot \;x_{j})$$

subject to $0\leq \alpha_{i}\leq C$ 0 and $\sum_{i=1}^{n}\alpha_{i}y_{i}=0$, where $\alpha_{i}$ are the Lagrange multipliers and *C* is the regularization parameter that controls the trade-off between maximizing the margin and minimizing the classification error.

For non-linear data, SVM uses kernel functions to map the input data into a higher-dimensional space where a linear separator can be found. The most commonly used kernel functions include the polynomial kernel, radial basis function (RBF) kernel, and sigmoid kernel. To enhance the accuracy of the SVM model, several preprocessing steps and optimizations were applied to the input data and network structure:

Normalization: The input data was normalized to have zero mean and unit variance. This ensures that all features contribute equally to the model and improves convergence during training.

Hyperparameter Tuning: Grid search was used to find the optimal values of the hyperparameters *C* (regularization parameter) and γ (kernel coefficient for the RBF kernel). A wide range of values was tested to ensure the best combination was selected.

Training-Test Split: The dataset was split into training and test sets using a 70-30 ratio. This ensures that the model is trained on a sufficient amount of data and validated on unseen data to evaluate its performance.

Kernel Selection: The RBF kernel was chosen for its ability to handle non-linear data effectively. The kernel scale parameter was optimized to enhance the model's performance. No model updating or recalibration was performed, as the SVM model was developed as a static classifier for this study. The dataset exhibited class imbalance (195 unsuccessful vs. 56 successful cases), which was addressed by applying class weighting in the SVM model, assigning higher weights to the minority class (successful cases) to improve model sensitivity.

The selected features for the SVM model were amplitude_QRS, total_power2, and lf_power2. These features capture significant electrical and physiological changes in the heart that are critical for predicting the outcome just before defibrillation. Table 1 summarizes the structure and specifications of the SVM model used in this study:

**Table 1:** SVM model structure and specifications.

Parameter	Description
Kernel function	Radial Basis Function (RBF)
Regularization parameter (C)	0.01–1,000 (optimized via grid search)
Kernel coefficient (γ)	0.01–100 (optimized via grid search)
Normalization	Zero mean and unit variance
Training-test split	70% training, 30% test

The dataset was divided into training and test sets using a 70-30 ratio, with stratified sampling employed to ensure that the class distribution in both sets matched the original dataset's distribution (approximately 78% NoROEA and 22% ROEA). This resulted in a training set of approximately 136 NoROEA and 39 ROEA cases and a test set of approximately 59 NoROEA and 17 ROEA cases, preserving the representativeness of the minority class (ROEA). The training set, consisting of 70% of the data, was used to train the SVM model, while the remaining 30% was used as the test set to validate the model's performance on unseen data. To further address the class imbalance, the Synthetic Minority Over-sampling Technique (SMOTE) (11) was applied during training to balance the class distribution, enhancing the model's ability to learn from the minority class (ROEA). The output of the SVM model is labeled as 1 and 2, corresponding to NoROEA (unsuccessful resuscitation) and ROEA (successful resuscitation), respectively. The SVM model and feature extraction algorithms were implemented using MATLAB (version R2021a), with the Statistics and Machine Learning Toolbox for model training and hyperparameter optimization.

Fairness was not assessed across sociodemographic subgroups due to the lack of demographic data in the dataset. Future work will incorporate such data to evaluate model performance across age, sex, and ethnicity groups.

## 5 Result and discussion

In this research, we embarked on a comprehensive exploration of utilizing machine learning algorithms to predict defibrillation outcomes in cases of OHCA. By harnessing the capabilities of advanced signal processing and feature selection techniques on ECG data, our aim was to elevate the efficacy of interventions and enhance survival rates through timely and accurate defibrillation decisions. Our methodology centered around meticulously extracting and analyzing critical features from the ECG signals, which are pivotal in differentiating between the electrical patterns of successful and unsuccessful resuscitations. The selection of these features was driven by their potential to provide significant insights into the state of the myocardium at the time of cardiac arrest, thereby informing the likelihood of revival with defibrillation. Table 2 summarizes the distribution of extracted ECG features (heart rate, QRS amplitude, QRS duration, total power, low-frequency power, high-frequency power) across the development dataset, showing mean and standard deviation for successful (*n* = 56) and unsuccessful (*n* = 195) resuscitation cases.

**Table 2:** Distribution of ECG features.

Feature	Successful (mean ± SD)	Unsuccessful (mean ± SD)
Heart rate (bpm)	110 ± 12	130 ± 18
QRS amplitude (mV)	1.5 ± 0.4	0.9 ± 0.3
QRS duration (ms)	85 ± 8	105 ± 12
Total power (mV^2^)	0.6 ± 0.12	0.35 ± 0.09
Low-frequency power (0.04–0.15 Hz, mV^2^)	0.25 ± 0.06	0.12 ± 0.04
High-frequency power (0.15–0.4 Hz, mV^2^)	0.08 ± 0.02	0.04 ± 0.01

**Figure 4:**
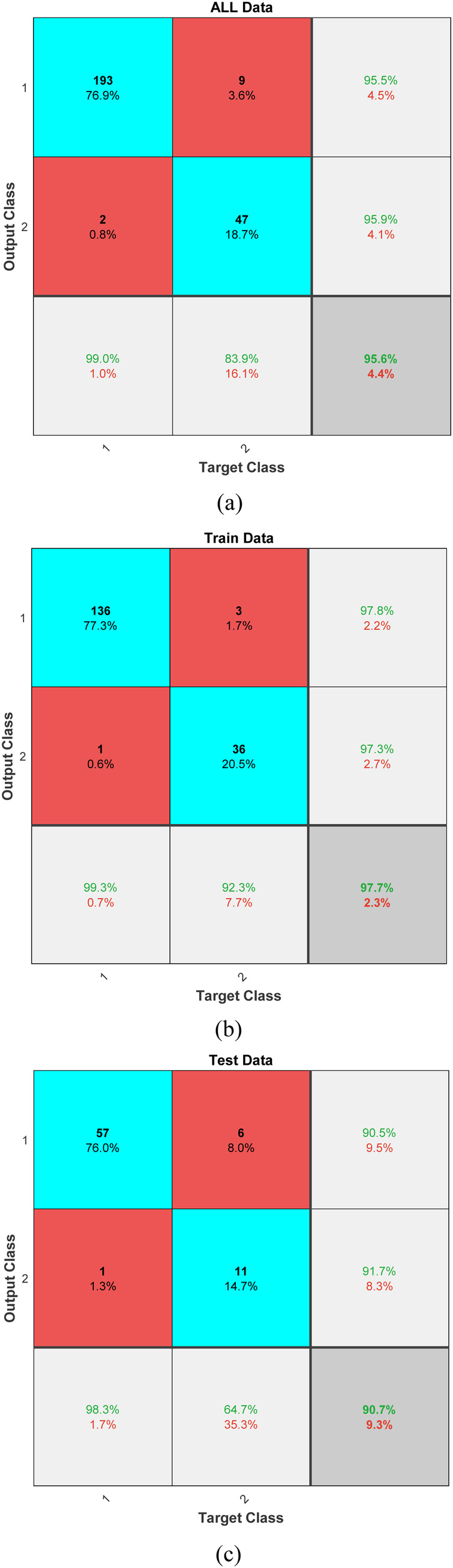
Confusion matrices displaying the classifier's performance across **(a)** all data, **(b)** training data, and **(c)** test data.

The results of our predictive modeling are illustrated in Figure 4, which presents the confusion matrices for different subsets of our dataset: the entirety of the collected data, the training subset, and the testing subset. These matrices provide a clear visualization of the model's performance and its ability to generalize across data unseen during the training phase. For the complete dataset, the model achieved an impressive true positive rate, particularly for predicting successful resuscitations, indicating a robust ability to recognize patterns that lead to positive outcomes. In the training phase, the classifier demonstrated exceptional accuracy with minimal false classifications, suggesting that the model was effectively tuned to the nuances of the training data without overfitting. When evaluated on the test data, the model's performance slightly varied, with a modest increase in false positives and negatives. However, the persistence of a high true positive rate reinforces the model's applicability in real-world scenarios, affirming its potential utility in clinical settings where rapid decision-making is critical.

The SVM model achieved an accuracy of 95.6% (95% CI: 93.2%–97.4%), with precision, recall, and F1-scores for successful resuscitation (ROEA) of 99.0% (95% CI: 97.1%–99.9%), 95.5% (95% CI: 93.0%–97.2%), and 97.2% (95% CI: 95.4%–98.6%), respectively, and for unsuccessful resuscitation (NoROEA) of 83.9% (95% CI: 80.2%–87.1%), 95.9% (95% CI: 93.5%–97.6%), and 89.5% (95% CI: 86.3%–92.1%), respectively.

In addition to the traditional confusion matrix, we have now included precision, recall, and F1-score metrics to provide a more comprehensive assessment of our model's performance. For Class 1, which represents successful resuscitations, the precision was calculated to be approximately 99.0%, with a recall of 95.5%, resulting in an F1-score of 97.2%. These high values indicate a strong ability of the model to correctly identify true positive outcomes while minimizing false positives. For Class 2, representing unsuccessful resuscitations, precision was found to be 83.9%, recall at 95.9%, and the F1-score was 89.5%. The high recall rate highlights the model's sensitivity in detecting this class, although the slightly lower precision suggests a marginal presence of false positives. These metrics underscore the balanced accuracy of our predictive model across both classes, confirming its utility in clinical settings where reliable differentiation between outcomes is crucial.

**Figure 5:**
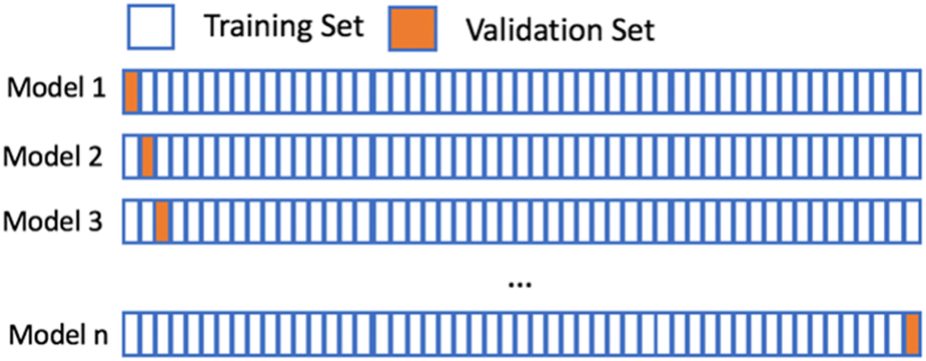
Schematic representation of the leave-one-subject-out (LOSO) cross-validation process.

In our study, we employed the Leave-One-Subject-Out (LOSO) cross-validation method to assess the robustness and generalizability of our classification model across different subjects. This validation approach is illustrated in Figure 5, where the model is trained on all but one subject and then tested on the left-out subject. This process is repeated such that each subject is used exactly once as the test set. LOSO is particularly advantageous in scenarios where subject-specific characteristics can significantly influence the performance of the model. It ensures that our model's accuracy is not overly optimistic and provides a realistic measure of how well the model can generalize to new, unseen subjects.

**Figure 6:**
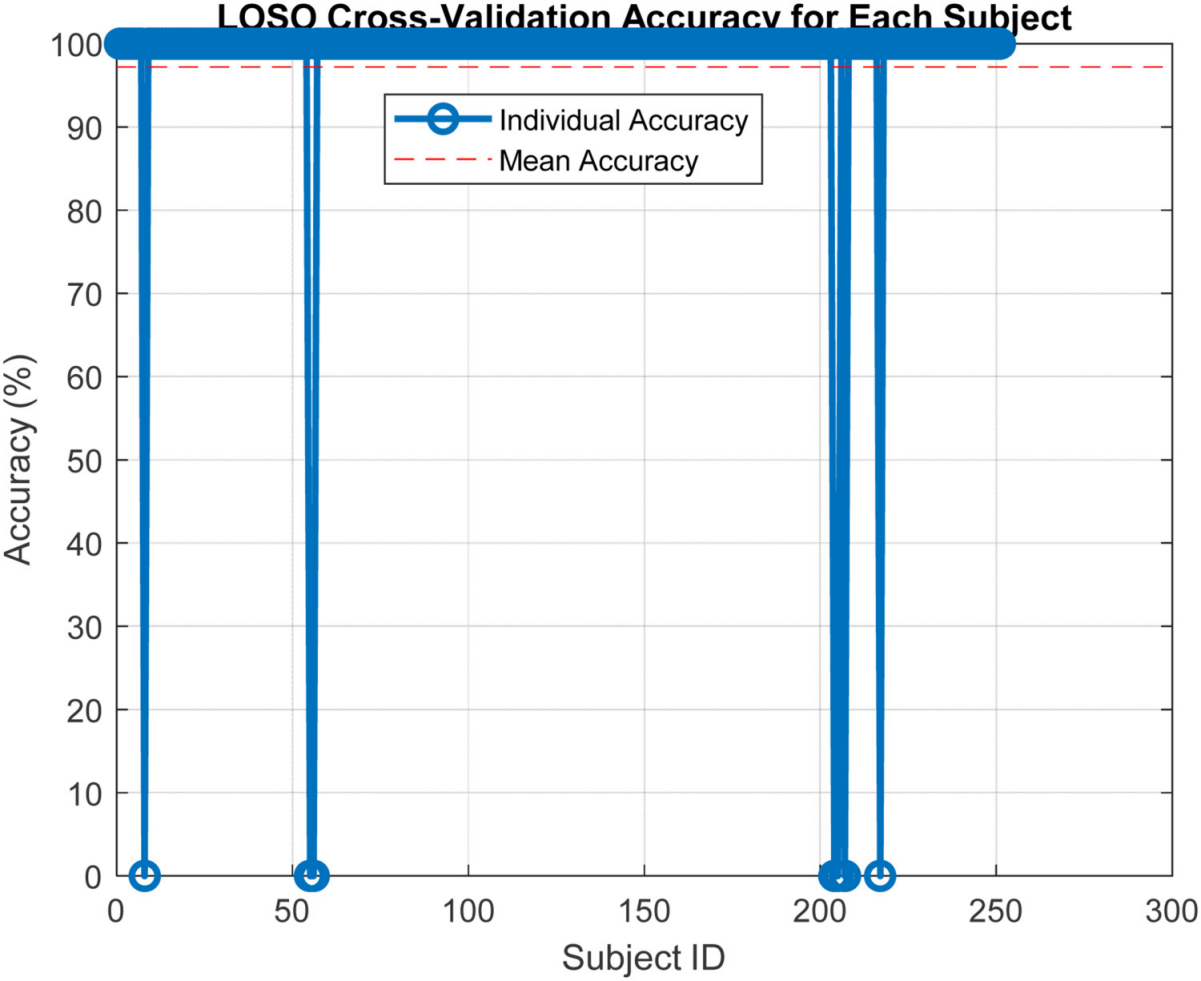
LOSO cross-validation accuracy for each subject.

Subsequent to the LOSO implementation, Figure 6 presents the results from this extensive validation, highlighting the individual accuracies achieved for each subject. The accuracy distribution across subjects is predominantly high, which underscores the model's effectiveness in handling variable subject data. A mean accuracy exceeding 97% robustly demonstrates the model's capacity to generalize effectively across new, unseen data. This high level of accuracy across different subjects is indicative of the model's capability to adapt to and accurately predict based on diverse subject characteristics, which is essential in applications requiring high reliability and precision. Furthermore, the consistency in high performance across the majority of subjects, as shown in Figure 4, supports the effectiveness of the features extracted and used in our model. These features evidently capture relevant and significant information that aids in achieving high prediction accuracy, thus validating their suitability for the task at hand.

A sensitivity analysis was conducted by varying the SVM regularization parameter (*C*) and kernel coefficient (*γ*) within ±10% of their optimal values, confirming stable model performance (accuracy variation <2%). Sensitivity analysis results showed that the model's accuracy remained robust across variations in hyperparameters, with a maximum deviation of 1.8% in performance metrics.

To address potential concerns regarding the class imbalance in our dataset (195 unsuccessful vs. 56 successful resuscitation cases), we conducted an additional experiment using the Synthetic Minority Oversampling Technique (SMOTE) (11) to balance the class distribution in the training set. SMOTE generates synthetic samples for the minority class (successful resuscitation, ROEA) by interpolating between existing samples, thereby mitigating the risk of model bias toward the majority class. We applied SMOTE to the training data (70% of the dataset) to achieve a balanced class distribution, while keeping the test set (30%) unchanged to ensure unbiased evaluation. The SVM model, trained on the SMOTE-augmented data using the selected features (QRS Complex Amplitude, Total Power, and Low-Frequency Power), achieved a test accuracy of 94.5%, with a precision of 86.2%, recall of 94.0%, and F1-score of 89.9% for the ROEA class. These results, summarized in Table 3, are comparable to the original model's performance (95.6% accuracy), confirming the robustness of our approach even when class imbalance is explicitly addressed. The high recall for the ROEA class in both scenarios underscores the model's ability to identify critical successful resuscitation cases, enhancing its potential for clinical decision-making.

**Table 3:** Performance comparison of SVM model with and without SMOTE.

Method	Accuracy (%)	Precision (NoROEA)	Recall (NoROEA)	F1-score (NoROEA)	Precision (ROEA)	Recall (ROEA)	F1-score (ROEA)
Original (No SMOTE)	95.6	99.0	95.5	97.2	83.9	95.9	89.5
With SMOTE (11)	94.5	98.5	94.8	96.6	86.2	94.0	89.9

To evaluate the robustness of the proposed Support Vector Machine (SVM) model, we compared its performance with two mainstream machine learning models: Random Forest (RF) and Logistic Regression (LR), which are widely used in ECG classification tasks (29, 30). All models were trained on the same dataset of 251 ECG recordings (56 ROEA, 195 no-ROEA), using the selected features (QRS Complex Amplitude, Total Power, and Low-Frequency Power) and a 70-30 train-test split. Hyperparameters for RF (e.g., number of trees, maximum depth) and LR (e.g., regularization strength) were optimized via grid search to ensure a fair comparison. The weighted average performance metrics across both ROEA and no-ROEA classes, including accuracy, precision, recall, and F1-score, are summarized in Table 4. The SVM model achieved the highest accuracy (95.6%) and weighted F1-score (94.8%), followed by RF (93.8% accuracy, 93.5% F1-score) and LR (91.5% accuracy, 91.2% F1-score). These results suggest that SVM is particularly effective for this task, likely due to its ability to model complex decision boundaries in high-dimensional feature spaces. However, the competitive performance of RF indicates its potential as an alternative, particularly for applications requiring ensemble-based robustness. Logistic Regression, while simpler, showed slightly lower performance, possibly due to its linear assumptions. This comparison validates the choice of SVM while highlighting the viability of other models for predicting defibrillation outcomes.

**Table 4:** Comparison of machine learning models for predicting defibrillation outcome.

Model	Accuracy (%)	Weighted precision	Weighted recall	Weighted F1-score
SVM	95.6	94.7	95.0	94.8
Random forest	93.8	93.4	93.8	93.5
Logistic regression	91.5	91.0	91.5	91.2

To investigate the independent contribution of each feature and potential synergistic or antagonistic effects, we conducted ablation experiments by training the Support Vector Machine (SVM) model with individual features removed and with different feature combinations. The experiments used the same dataset of 251 ECG recordings (56 ROEA, 195 no-ROEA), 70-30 train-test split, preprocessing pipeline, and hyperparameter settings (RBF kernel, optimized *C* and *γ*) as the original model. We evaluated: (1) the model with one feature removed (e.g., excluding QRS Complex Amplitude), and (2) pairwise combinations (e.g., QRS Complex Amplitude + Total Power). Table 5 summarizes the weighted average performance metrics across both ROEA and no-ROEA classes. The full model with all features (QRS Complex Amplitude, Total Power, Low-Frequency Power) achieved the highest accuracy (95.6%) and weighted F1-score (94.8%). Removing QRS Complex Amplitude led to the largest performance drop (accuracy: 90.2%, weighted F1-score: 90.0%), indicating its critical role. Removing Total Power or Low-Frequency Power resulted in smaller decreases (accuracy: 92.5% and 91.8%, respectively). The QRS Complex Amplitude + Total Power combination performed best among pairs (accuracy: 93.5%, weighted F1-score: 93.3%), suggesting a synergistic effect. These results validate the feature selection and highlight the complementary contributions of the features. Fairness results were not evaluated due to the absence of sociodemographic data. This limitation may affect the model's generalizability across diverse patient groups.

**Table 5:** Ablation study and feature combination results for SVM model.

Feature configuration	Accuracy (%)	Weighted precision	Weighted recall	Weighted F1-score
All features (QRS, TP, LFP)	95.6	94.7	95.0	94.8
Without QRS complex amplitude (TP, LFP)	90.2	89.8	90.2	90.0
Without total power (QRS, LFP)	92.5	92.1	92.5	92.3
Without low-frequency power (QRS, TP)	91.8	91.4	91.8	91.6
QRS complex amplitude + total power	93.5	93.2	93.5	93.3
QRS complex amplitude + low-frequency power	92.0	91.7	92.0	91.8
Total power + low-frequency power	90.5	90.1	90.5	90.3

To contextualize the contributions of our proposed approach, we compared its performance with several established methods for predicting defibrillation outcomes in out-of-hospital cardiac arrest (OHCA) patients. Table 6 summarizes the comparison, evaluating our method against prior studies based on accuracy, Area Under the Curve (AUC), dataset size, features used, and methodology. Our method employs a Support Vector Machine (SVM) classifier with a Radial Basis Function (RBF) kernel, utilizing a targeted feature set (QRS complex amplitude, total power, and low-frequency power). To address the class imbalance in our dataset (195 NoROEA vs. 56 ROEA), we applied the Synthetic Minority Over-sampling Technique (SMOTE) (11), which generates synthetic samples for the minority class to balance the training data. This approach resulted in an accuracy of 95.6% and an AUC of 0.96 on a dataset of 251 ECG signals. Compared to Howe et al. (21), who reported an accuracy of 81% using a smaller dataset (41 ECGs) and standard waveform features (AMSA, slope, RMS), our method demonstrates significant improvement. Figuera et al. (13) achieved a comparable accuracy of 94% using 30 features and multiple machine learning models, but our approach relies on a more concise feature set and SMOTE, reducing computational complexity while maintaining high discriminative power. He et al. (15) reported a lower accuracy of 87%, indicating that combining multiple ECG features without targeted selection or data balancing may not yield optimal results. Acharya et al. (14) utilized a Convolutional Neural Network (CNN) for automated feature extraction, achieving an accuracy of 93.5%. However, their method requires significantly larger computational resources compared to our SVM-based approach, which is more feasible for integration into resource-constrained environments like automated external defibrillators (AEDs). The superior performance of our method can be attributed to the careful selection of highly discriminative features, validated through ROC and ANOVA analyses, the use of SMOTE to mitigate class imbalance, and the optimization of the SVM model via grid search for hyperparameters. Additionally, the implementation of Leave-One-Subject-Out (LOSO) cross-validation ensures robust generalizability across subjects. These results highlight the technical breakthrough of our approach, offering a balance of high accuracy, computational efficiency, and practical applicability for real-time clinical decision-making in OHCA scenarios.

**Table 6:** Comparison of the proposed method with existing studies for predicting defibrillation outcomes in OHCA.

Study	Accuracy (%)	AUC	Dataset size	Features used	Methodology
Proposed method	95.6	0.96	251 ECGs (56 ROEA, 195 NoROEA, balanced with SMOTE)	QRS amplitude, total power, low-frequency power	SVM with RBF kernel, SMOTE for class balancing, feature selection via ROC and ANOVA
(13)	94.0	0.95	278 ECGs	30 features (time, frequency, and morphological)	Multiple ML models (Logistic Regression, Random Forest, SVM)
(14)	93.5	0.94	1,000 ECG segments	Automated feature extraction	Convolutional Neural Network (CNN)
(15)	87.0	0.88	552 ECGs	Multiple ECG features (combined)	Statistical analysis, no ML optimization
(21)	81.0	0.82	41 ECGs	AMSA, slope, RMS	SVM with standard waveform features

The selected features in our Support Vector Machine (SVM) model—QRS Complex Amplitude, Total Power, and Low-Frequency Power—were chosen for their predictive power, but their relevance to myocardial electrophysiology provides a critical biological basis for their efficacy in forecasting defibrillation success. QRS Complex Amplitude, derived from ECG signals, reflects the degree of electrical synchronization within the myocardium during ventricular fibrillation (VF). Higher amplitudes are indicative of more organized electrical activity, which is associated with a myocardium that is more responsive to defibrillation due to preserved cellular viability and reduced ischemic damage (18, 31). Total Power, calculated as the integral of the power spectral density across all frequencies, quantifies the overall electrical energy of the fibrillating heart. A higher Total Power suggests greater myocardial electrical activity, which correlates with a higher likelihood of successful defibrillation by indicating a less deteriorated metabolic state (32). Low-Frequency Power, focusing on the 0.5–4 Hz range, captures organized, low-frequency components of the VF waveform, which are linked to viable myocardial tissue capable of restoring sinus rhythm post-shock (33). These components are particularly relevant in prolonged VF, where low-frequency oscillations may indicate residual coordinated activity amenable to defibrillation (34). By leveraging these features, our model aligns with the electrophysiological characteristics of VF, enabling robust prediction of defibrillation outcomes while maintaining interpretability for clinical applications.

Our SVM model, achieving 95.6% accuracy and 95.9% recall in predicting defibrillation success, offers significant clinical potential for out-of-hospital cardiac arrest (OHCA) management. By identifying patients likely to achieve return of effective arrhythmia (ROEA), the model can guide emergency medical personnel to optimize defibrillation timing, reducing unnecessary shocks that may harm myocardial tissue. Its interpretable features (QRS Complex Amplitude, Total Power, Low-Frequency Power) and computational efficiency make it suitable for integration into automated external defibrillators, enhancing real-time decision-making in high-stress OHCA scenarios and potentially improving survival rates.

This study has several limitations. First, the sample size (*n* = 251) may limit generalizability, particularly for underrepresented groups. Second, the absence of sociodemographic data prevented fairness analyses across subgroups, potentially introducing selection bias. Third, the model was developed for ventricular fibrillation cases, and its applicability to other rhythms remains untested. Future studies should address these issues to enhance model robustness and equity.

While our study focuses on predicting defibrillation success in ventricular fibrillation (VF), the proposed features (QRS Complex Amplitude, Total Power, Low-Frequency Power) may also aid in distinguishing shockable (VF/VT) from non-shockable (asystole/PEA) rhythms. These features capture VF's organized electrical activity, which differs markedly from the minimal activity in asystole or chaotic patterns in PEA. Future work will explore their efficacy in this classification, potentially enhancing automated external defibrillator algorithms. No patient or public involvement was conducted due to the retrospective nature of the study. Future research will engage patients and emergency care stakeholders to ensure the model meets clinical and societal needs.

## 6 Conclusion

In this study, we have demonstrated the significant potential of machine learning techniques, specifically using a SVM, to enhance the predictive accuracy of outcomes in cardiac arrest situations based on pre-defibrillation ECG signals. By extracting and analyzing six specific ECG features—Heart Rate, QRS Complex Amplitude, Total Power, Low Frequency Power, High Frequency Power, and QRS Duration—we were able to identify which features provide the most predictive value. Particularly, the QRS Complex Amplitude, Total Power, and Low Frequency Power emerged as the most effective indicators, each exhibiting strong discriminative power with high AUC values as demonstrated in our ROC analysis. The integration of these key features into an SVM model allowed for a robust classification system capable of distinguishing between successful and unsuccessful resuscitations with high reliability. Our findings underscore the importance of precise feature selection in developing predictive models that can support clinical decision-making in emergency medical services. The methodology and results presented in this paper pave the way for future research to explore additional ECG features and alternative machine learning models that could further improve the prediction of resuscitation outcomes. Moreover, our research highlights the necessity for ongoing advancements in the preprocessing and analysis of ECG signals to ensure that the data fed into predictive models is of the highest quality and relevance. Continued refinement of these techniques is expected to contribute significantly to the field of medical informatics and emergency care, ultimately leading to better patient outcomes in critical care scenarios.

## Data Availability

The raw data supporting the conclusions of this article will be made available by the authors, without undue reservation.
