# Supplementary material for: Advanced ECG feature extraction and SVM classification for predicting defibrillation success in OHCA
Source: Front Cardiovasc Med. 2025 Jul 16;12:1550422. doi: 10.3389/fcvm.2025.1550422 (PMC12308557; doi:10.3389/fcvm.2025.1550422)
Supplement: Supplementary file 1 [file Table1.docx]

**TRIPOD+AI Checklist for Manuscript Compliance**

| **Item No.** | **Description** | **Page/Section in Manuscript** | **Status** | **Notes** |
| --- | --- | --- | --- | --- |
| 1 | Title: Identify the study as developing or evaluating a multivariable prediction model | Page 1, Title | Covered | Title specifies model development for defibrillation success in OHCA using SVM. |
| 2 | Abstract: See TRIPOD+AI for Abstracts checklist | Page 1, Abstract | Covered | Includes objectives, methods, data, results. Verify against TRIPOD+AI Abstracts checklist (not provided). |
| 3a | Background: Explain the healthcare context and rationale for the model | Pages 2–5, Introduction | Covered | Discusses OHCA context and references existing models ([10, 13, 15, 21]). |
| 3b | Describe model identification in the care pathway, including intended users | Page 2, Introduction | Covered | Specifies use by emergency physicians and paramedics in OHCA care pathway. |
| 3c | Describe known health inequalities between sociodemographic groups | Page 5 or 12, Introduction or Discussion | Covered | Notes lack of demographic data and need for future fairness analysis. |
| 4 | Objectives: Specify study objectives (development or evaluation) | Page 5, Introduction | Covered | States development of SVM model for defibrillation success prediction. |
| 5a | Data: Describe data sources for development and evaluation | Page 6, Data Acquisition | Covered | Describes 251 ECG signals from Benini et al. [22]. |
| 5b | Specify eligibility criteria for participants | Page 6, Data Acquisition | Covered | Specifies inclusion (VF, 9-second ECG) and exclusion (incomplete/non-VF signals). |
| 5c | Report number of participants and outcome events | Page 6, Data Acquisition | Covered | Reports 251 patients (56 ROEA, 195 NoROEA). |
| 6 | Define the outcome and measurement method | Page 6, Data Acquisition | Covered | Outcome (ROEA/NoROEA) defined by cardiologists per [22]. |
| 7 | Define predictors and measurement methods | Pages 7–8, Data Processing | Covered | Six ECG features and extraction methods described. |
| 8a | Describe data preprocessing methods | Pages 6, 9, Data Acquisition, SVM | Covered | Details resampling, filtering, and normalization. |
| 8b | Describe predictor selection methods | Page 8, Data Processing | Covered | Feature selection via ROC and ANOVA. |
| 9a | Describe sampling method for model development | Page 10, SVM | Covered | 70-30 train-test split with stratified sampling. |
| 9b | Describe handling of missing data | Page 7, Data Processing | Covered | States no missing data in 251 signals. |
| 9c | Detail modeling method (e.g., algorithm, hyperparameters) | Pages 9–10, SVM | Covered | SVM with RBF kernel, grid search, and SMOTE described. |
| 10a | Specify performance measures (development) | Page 11, Results | Covered | Reports accuracy (95.6%), precision, recall, F1-score, confusion matrices. |
| 10b | Specify evaluated models and performance measures | N/A | Not Applicable | Focuses on development, not external evaluation. |
| 11 | Describe validation methods and iterations | Page 12, Results | Covered | LOSO cross-validation with mean accuracy >97%. |
| 12a | Specify software and packages used | Page 9, SVM | Covered | Specifies MATLAB R2021a and Statistics Toolbox. |
| 12b | Describe model updating or recalibration | Page 9, SVM | Covered | States no updating performed. |
| 12c | Describe methods for comparing models | Page 13, Results | Covered | Compares SVM with Random Forest and Logistic Regression. |
| 12d | Describe participant flow | Page 6, Data Acquisition | Covered | Describes exclusion of 49 cases from 300, with flow diagram reference. |
| 12e | Describe distribution of predictors and outcomes | Page 10, Results | Covered | Table 2 shows feature distributions. |
| 12f | Report class imbalance and handling methods | Pages 10, 13, SVM, Results | Covered | Details SMOTE [11] for class imbalance (195 vs. 56). |
| 13 | Describe methods for assessing fairness in subgroups | Page 9 or 12, SVM or Discussion | Covered | Notes lack of fairness analysis due to missing demographic data. |
| 14 | Describe sensitivity or robustness analyses | Page 11, Results | Covered | Describes hyperparameter variation analysis. |
| 15 | Describe study population characteristics | Page 10, Results | Covered | Paragraph describes 251 patients and lack of demographic data. |
| 16 | Results: Present final model | Page 9, SVM | Covered | SVM specifications in Table 1. |
| 17a | Report performance measures | Pages 11–13, Results | Covered | Accuracy, precision, recall, F1-score, and LOSO results reported. |
| 17b | Report uncertainty in performance measures | Page 12, Results | Covered | Confidence intervals added for performance metrics. |
| 18 | Report fairness results | Page 11 or 12, Results or Discussion | Covered | Notes no fairness results due to missing demographic data. |
| 19a | Report sensitivity analysis results | Page 11, Results | Covered | Reports hypothetical hyperparameter variation results. |
| 19b | Report model comparison results | Page 13, Results | Covered | Comparison with Random Forest and Logistic Regression. |
| 20 | Interpret results and compare with prior studies | Pages 11–15, Results | Covered | Compares results with [13, 14, 15, 21]. |
| 21 | Discuss clinical and operational implications | Page 15, Results | Covered | Discusses AED integration and OHCA decision-making. |
| 22 | Discuss limitations (e.g., bias, generalizability) | Page 16, Conclusions | Covered | Expanded to include sample size, fairness, and VF focus limitations. |
| 23 | Discuss future directions | Page 16, Conclusions | Covered | Suggests dataset expansion and model exploration. |
| 24 | Report patient and public involvement | Page 6, Data Acquisition | Covered | States no PPI due to retrospective study. |
| 25a | Report funding sources | Page 19, End | Covered | Confirms no external funding. |
| 25b | Report conflicts of interest | Page 19, End | Covered | States no conflicts of interest. |
| 25c | Report access to study protocol | Page 19, End | Covered | States protocol unavailability due to retrospective study. |
| 25d | Report study registration | Page 19, End | Covered | States no registration due to retrospective study. |
| 25e | Report data sharing | Page 19, End | Covered | References Benini et al. [22] with access restrictions. |
| 25f | Report code sharing | Page 19, End | Covered | Confirms code in Supplementary File 1. |
